# A 3D-Printed Biomaterial Scaffold Reinforced with Inorganic Fillers for Bone Tissue Engineering: In Vitro Assessment and In Vivo Animal Studies

**DOI:** 10.3390/ijms24087611

**Published:** 2023-04-20

**Authors:** Mduduzi N. Sithole, Pradeep Kumar, Lisa C. Du Toit, Kennedy H. Erlwanger, Philemon N. Ubanako, Yahya E. Choonara

**Affiliations:** 1 Wits Advanced Drug Delivery Platform Research Unit, Department of Pharmacy and Pharmacology, School of Therapeutic Sciences, Faculty of Health Sciences, University of the Witwatersrand, 7 York Road, Parktown, Johannesburg 2193, South Africa; 2 School of Physiology, Faculty of Health Sciences, University of the Witwatersrand, 7 York Road, Parktown, Johannesburg 2193, South Africa

**Keywords:** osteoblast-like cells, 3D printing, alginate, inorganic fillers, bone regeneration

## Abstract

This research aimed to substantiate the potential practicality of utilizing a matrix-like platform, a novel 3D-printed biomaterial scaffold, to enhance and guide host cells’ growth for bone tissue regeneration. The 3D biomaterial scaffold was successfully printed using a 3D Bioplotter^®^ (EnvisionTEC, GmBH) and characterized. Osteoblast-like MG63 cells were utilized to culture the novel printed scaffold over a period of 1, 3, and 7 days. Cell adhesion and surface morphology were examined using scanning electron microscopy (SEM) and optical microscopy, while cell viability was determined using MTS assay and cell proliferation was evaluated using a Leica microsystem (Leica MZ10 F). The 3D-printed biomaterial scaffold exhibited essential biomineral trace elements that are significant for biological bone (e.g., Ca-P) and were confirmed through energy-dispersive X-ray (EDX) analysis. The microscopy analyses revealed that the osteoblast-like MG63 cells were attached to the printed scaffold surface. The viability of cultured cells on the control and printed scaffold increased over time (*p* < 0.05); however, on respective days (1, 3, and 7 days), the viability of cultured cells between the two groups was not significantly different (*p* > 0.05). The protein (human BMP-7, also known as growth factor) was successfully attached to the surface of the 3D-printed biomaterial scaffold as an initiator of osteogenesis in the site of the induced bone defect. An in vivo study was conducted to substantiate if the novel printed scaffold properties were engineered adequately to mimic the bone regeneration cascade using an induced rabbit critical-sized nasal bone defect. The novel printed scaffold provided a potential pro-regenerative platform, rich in mechanical, topographical, and biological cues to guide and activate host cells toward functional regeneration. The histological studies revealed that there was progress in new bone formation, especially at week 8 of the study, in all induced bone defects. In conclusion, the protein (human BMP-7)-embedded scaffolds showed higher regenerative bone formation potential (week 8 complete) compared to the scaffolds without protein (e.g., growth factor; BMP-7) and the control (empty defect). At 8 weeks postimplantation, protein (BMP-7) significantly promoted osteogenesis as compared to other groups. The scaffold underwent gradual degradation and replacement by new bones at 8 weeks in most defects.

## 1. Introduction

Bone defects are among the main cause of morbidity and disability in elderly patients [1]. Bone tissues have a natural ability to self-regenerate or self-heal; however, large-sized (critical-sized: 5 mm in diameter) bone defects are reported to not self-heal to completion without external intervention [2,3]. Medical therapy for repairing/regenerating bone defect tissue remains a challenge; hence, this is one of the major focuses for bone tissue engineering research [4]. Advanced approaches in bone tissue engineering application are an universal clinical necessity to regenerate/repair/restore critical-sized bone defects originating from various pathological conditions or trauma [5,6]. The development of methods and biomaterial systems that can permanently and perfectly repair/regenerate damaged bone tissue are of great significance for clinical use in patients [4]. The developed 3D biomaterial scaffold systems over the years have shown great potential as solutions for repairing/regenerating bone tissue defects [3]. However, there is still a huge need to fabricate 3D biomaterial scaffold systems interventions with appropriate properties for bone tissue engineering applications.

Generally, the 3D biomaterial scaffold should have similar mechanical support as the native tissue of interest (e.g., bone tissue), and must be easily resorbed while the new bone tissue forms [7]. The selection of biomaterials with appropriate properties for the formation of 3D biomaterial scaffold is significant, since they facilitate resorption and stimulate native in vivo tissue in the body to self-heal [8]. In this research article, biomaterials refers to materials that interface with biological systems [9,10,11,12]. Furthermore, the bonding strength at the scaffold interface has to be considered when choosing the biomaterials [13]. The most investigated natural polymers for bone tissue engineering applications include, but are not limited to, alginate, hyaluronic acid, chitosan, silk, and collagen [14]. However, natural polymers have limitations when used alone for the fabrication of bone tissue engineering biomaterial scaffolds [15], such as poor mechanical properties, which are suboptimal, amongst others [16,17].

Alginate, as a natural biomaterial polymer, is highly used and extensively studied, having a variety of applications in tissue engineering (e.g., wound healing and bone regeneration). Alginate is a non-toxic biomaterial that can form a biocompatible gel or hydrogel [18,19,20]. Alginate hydrogels exhibit poor mechanical properties, and due to their inert character, cells do not bind on its surface; in other words, alginate does not offer a binding site for cells [21,22]. Due to these limitations, composite biomaterials can be explored to circumvent the limitations, that is, by combining alginate either with inorganic fillers or polymers (e.g., Polyethylenimine (PEI)) or both [18,23]. The use of inorganic fillers to develop composite biomaterial scaffolds to improve the mechanical properties of the scaffolds is the main focus in different fields of research [24,25]. Therefore, a variety of inorganic fillers have been used to enhance relevant biomaterial scaffold properties for the tissue of interest [26,27,28]. The most utilized inorganic fillers include metallic particles such as gold nanoparticles [29] or hydroxyapatite [30], cellulose nanofibers [31,32], or bioactive glass (BG) [33,34]. In our laboratory, Sithole et al. (2018) used a one-step approach, by using hydrogel–filler composites for 3D printing a biomaterial scaffold, where the inorganic filler (silica gel powder) was directly incorporated into the alginate-based ink (hydrogel) to alter the rheological properties for improved shape fidelity, cell proliferation and differentiation [35]. In this study, we further examined the in vivo potential of the 3D-printed biomaterial scaffold in an induced critical-sized bone defect.

Furthermore, osteoblast-like cells were introduced into the 3D biomaterial scaffold in order to generate ex vivo tissue constructs to be used for biocompatibility and cell response (cell adhesion, cell proliferation, and cell viability) investigations [36,37,38,39,40]. Hence, osteoblast-like MG63 cells from human osteosarcoma were used in this study since they are reported to be anchorage-dependent. Supporting structures such as 3D biomaterial scaffolds are significant in acting as a template for the cells to grow, proliferate, and adhere [41]. This research article presents the fabrication of a composite 3D-printed biomaterial scaffold [35]. The 3D-printed biomaterial scaffold was evaluated using techniques such as scanning electron microscopy (SEM), optical microscopy, energy-dispersive X-ray (EDX) analysis, and the Leica microsystem (Leica MZ10 F). In vitro studies were conducted using the osteoblast-like MG63 cells to evaluate cellular response (such as cell adhesion, proliferation, and viability) followed by in vivo study utilizing the New Zealand White Rabbit model. The 3D-printed biomaterial scaffolds were implanted in the induced nasal bone defects of New Zealand White Rabbits to investigate the potential progress of bone tissue regeneration/formation. The in vivo assessments allow for the clinical translation of the novel 3D-printed biomaterial scaffold intervention to be subjected to potential realistic conditions [42].

Experimental animal models have been developed and used as an effective platform to explore the therapeutic potential of different developed novel therapeutic systems [43]. This is in part because most animals share both physiological and genetic similarities with humans, and this aids in assessing the clinical translation potential of the therapeutic agent being investigated for later clinical studies [43,44]. Therefore, this research evaluated the potential effectiveness and feasibility of the novel 3D-printed biomaterial scaffolds on induced critical-sized bone defects in rabbits.

## 2. Results

A 3D biomaterial scaffold was successfully printed employing an in situ conjugation–fabrication approach whereby the conjugation was achieved between the hydrogel-ink (alginate-based) and forming solution (Polyethylenimine (PEI) in ethanol). The fabrication part was performed employing a 3D BioPlotter^®^ (EnvisionTEC, GmBH, Gladbeck, Germany) and the biomaterial scaffolds were designed to fit the deformations created within the in vivo studies. An important aspect of this study was to evaluate the in vitro (e.g., cell adhesion, proliferation, and viability) and the potential in vivo (bone tissue regeneration) of the novel 3D-printed biomaterial scaffold in New Zealand White Rabbits.

### 2.1. Complexation and Gelation Kinetics of the Biomaterial Hydrogels (Hydrogel-Inks)

**Figure 1:**
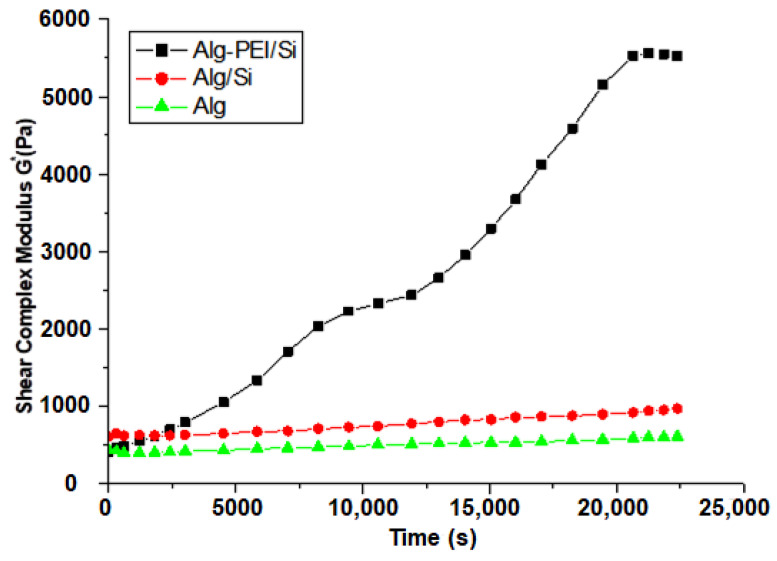
Complexation and gelation kinetics of the biomaterials (Alg, AlgSi, and Alg-PEI/Si), with each data point representing the mean (*n* = 3).

Biomaterial hydrogels’ complexation and gelation kinetics are significant parameters to be investigated before the 3D printing application. This is to have a clear understanding of the appropriate time it may take to print the next layer without any deviations and to confirm the ionic interaction between the two ionic biomaterials during the printing process. Figure 1 depicts the gelation kinetics (e.g., the speed of hardening and complexation) of the biomaterial hydrogels (hydrogel-inks). The result shows that the two (alginate/silica gel powder (Alg/Si) and alginate (Alg)) procured biomaterial hydrogels (hydrogel-inks) never changed their mechanical properties (that includes gelation, mechanical strength, etc.); hence, it was concluded that there was no ionic interaction among other interactions. However, for the formation of the hybrid alginate–polyethylenimine/silica gel powder (Alg-PEI/Si) complex, there was an ionic interaction evident from the increased shear complex modulus (Pa) leading to increased gelation time. These are good properties for the appropriate gelation time necessary for 3D printing of these particular ionic biomaterial inks.

### 2.2. Preliminary Biomechanical Evaluation of 3D-Printed Biomaterial Scaffolds from Different Inorganic Fillers

A preliminary mechanical comparison investigation was conducted, and Table 1 provides the determined results of the preliminary investigations for Young’s modulus of the three different impregnated inorganic fillers (silica gel powder (Si), hydroxyapatite (HAP), and nanoclay) into the 3D-printed alginate-based scaffolds. The Alg-PEI/Si 3D-printed biomaterial scaffold, Alg-PEI/HAP 3D-printed biomaterial scaffold, and Alg-PEI/nanoclay 3D-printed biomaterial scaffold were evaluated to determine the Young’s modulus that is most preferred for bone tissue engineering application. Hence, the preferred 3D-printed biomaterial scaffold composite was identified and subjected to further characterization. Therefore, the 3D-printed biomaterial scaffold impregnated with silica gel powder (Si) (Alg-PEI/Si) was identified (depicted in Table 1) as the preferred scaffold for potential bone tissue application, since it showed a higher Young’s modulus of 60 MPa compared to nanoclay with 30 MPa Young’s modulus and 10 MPa Young’s modulus of HAP. The attained Young’s modulus of 60 MPa shows an improved alginate hybrid scaffold strength for bone tissue engineering application which is within acceptable values reported for cancellous bone repair (~20–500 MPa) [45]. This highlighted that the 3D-printed biomaterial scaffold’s current form possesses mechanical abilities for a particular bone tissue engineering application (e.g., cancellous bone repair).

### 2.3. Surface Morphology, Interconnectivity, Pore Size, Porosity, and Biomineralization Evaluation of the 3D-Printed Biomaterial Scaffold

**Figure 2:**
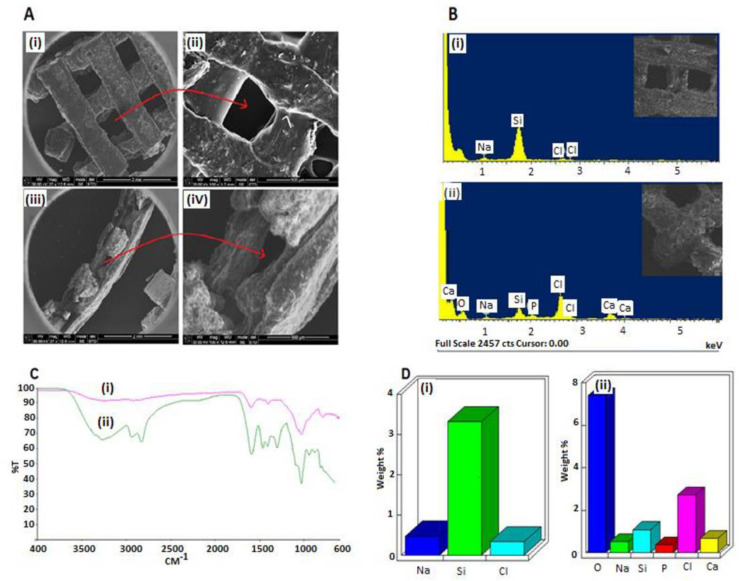
The cross-section and biomineralization investigation of the printed scaffold where (**A**) depicts the SEM (**i**) image of the surface section of the printed scaffold, (**ii**) magnified image of the surface section of the printed scaffold, (**iii**) image of the cross-section of the printed scaffold, and (**iv**) magnified image of the cross-section of the printed scaffold, (**B**) depicts the EDX analysis for (**i**) scaffold before immersion (non-mineralized) and (**ii**) after immersion in SBF (pH 7.4, 37 °C), (**C**) depicts the FT-IR spectra for (**i**) non-mineralized and (**ii**) mineralized scaffold, and (**D**) depicts the quantitative results of (**i**) non-mineralized and (**ii**) mineralized.

The 3D printing technology has the potential to control the scaffold’s pore size and design [46]. The SEM of the 3D-printed biomaterial scaffold in Figure 2(Ai–iv) provides the morphological surface, interconnectivity, and porous nature of the 3D-printed biomaterial scaffold with consistent channeling of pores, which are favorable for nutrient flow and material transfer, as well as providing a large surface area for attachment and proliferation of cells. Figure 2(Aiii,iv) depicts the cross-section part of the novel 3D-printed biomaterial scaffold, highlighting the pore interconnectivity of the scaffold, which is also good for the transportation of nutrients and minerals, amongst others. Pores’ interconnectivity within the 3D-printed biomaterial scaffold is a significant parameter, influencing cell seeding, cell adhesion, cell migration, and cell ingrowth that are necessary for promoting tissue regeneration in three dimensions. Scaffold pore size and porosity not only affect the cell behavior and its potential to differentiate, but they also affect the scaffold’s mechanical properties [47]. Therefore, the current 3D-printed biomaterial scaffold has a higher Young’s modulus of 60 MPa compared to the previous 3D-printed biomaterial scaffold by Sithole et al. [35], which was 18 MPa. This difference was due to the pore size of the current scaffold, since it was smaller (210 ± 10 µm) compared to the previous 3D-printed biomaterial scaffold pore size of 360 ± 20 µm. Therefore, it was observed that as the pore size decreased, Young’s modulus of the 3D-printed biomaterial scaffold increased and vice versa [35].

Figure 2(Aii) depicts the surface roughness of the novel 3D-printed biomaterial scaffold. The scaffold’s surface roughness is among the significant parameters that influence cell behavior and cell adhesion. The surface roughness of materials modulates the biological response of the tissues in contact with it and directly influences the in vitro as well as in vivo cellular morphology, phenotype expression, and proliferation [48,49]. Cells growing on the rough surface are stimulated towards differentiation as compared to cells growing on a smooth surface, as shown by their gene expression as reported in the literature [50].

There are many essential trace elements present in biological bone, including sodium (Na), chlorine (Cl), potassium (K), silicon (Si), and calcium (Ca), which play a significant role in bone growth. However, studies have shown that osteogenesis and angiogenesis can be induced by silicon ions, amongst other elements [51]. Surface biomineralization analysis of the 3D-printed biomaterial scaffold was performed using EDX and FT-IR characterization techniques for 3D-printed biomaterial scaffolds that were immersed in simulated body fluid (SBF) (pH 7.4; 37 °C) for 7 days. Our investigation showed that the 3D-printed biomaterial scaffolds showed potential biomineralization abilities when immersed in SBF. Figure 2(Bi,Di) depicts a pictorial EDX analysis of the 3D-printed biomaterial scaffold before being immersed in SBF, and Figure 2(Bii,Dii) depicts the EDX analysis after being immersed in SBF. Hence, the EDX analysis confirmed the presence of elements such as chlorine (Cl), calcium (Ca), sodium (Na), silicon (Si), and phosphorus (P) in the mineralized (immersed in SBF) scaffold (Figure 2(Bii,Dii)). The control scaffold (Figure 2(Bi,Di)) showed the presence of only three elements, which were chlorine (Cl), sodium (Na), and silicon (Si).

Furthermore, Figure 2(Ci) depicts the FTIR spectra of the scaffolds prior to being immersed in SBF, and Figure 2(Cii) depicts FTIR spectra after being immersed in SBF. Therefore, Figure 2(Ci) shows the gradual disappearance of or intensity decrease in certain spectrum absorption of the scaffold after immersion in SBF (pH 7.4; 37 °C). Despite these noticeable changes in the two spectra, Figure 2(Ci) shows characteristic bands such as the vibrational bands at 1590 cm^−1^ and 1407 cm^−1^, which are partially assigned to CO_2_^3−^, and the vibration bands observed at 1028 cm^−1^ and 785 cm^−1^ are in part attributed to PO_4_^3−^ groups. There was gradual precipitation of the calcium phosphate layer on the surface of the scaffold indicative of the biomineralization of our implant. Hence, the EDX and FTIR analysis further confirmed the 3D-printed biomaterial scaffold with exceptional biominerals on its surface, which are excellent for bone tissue engineering applications.

### 2.4. Evaluation of Cell Adhesion and Cell Confluence on the 3D-Printed Biomaterial Scaffold

**Figure 3:**
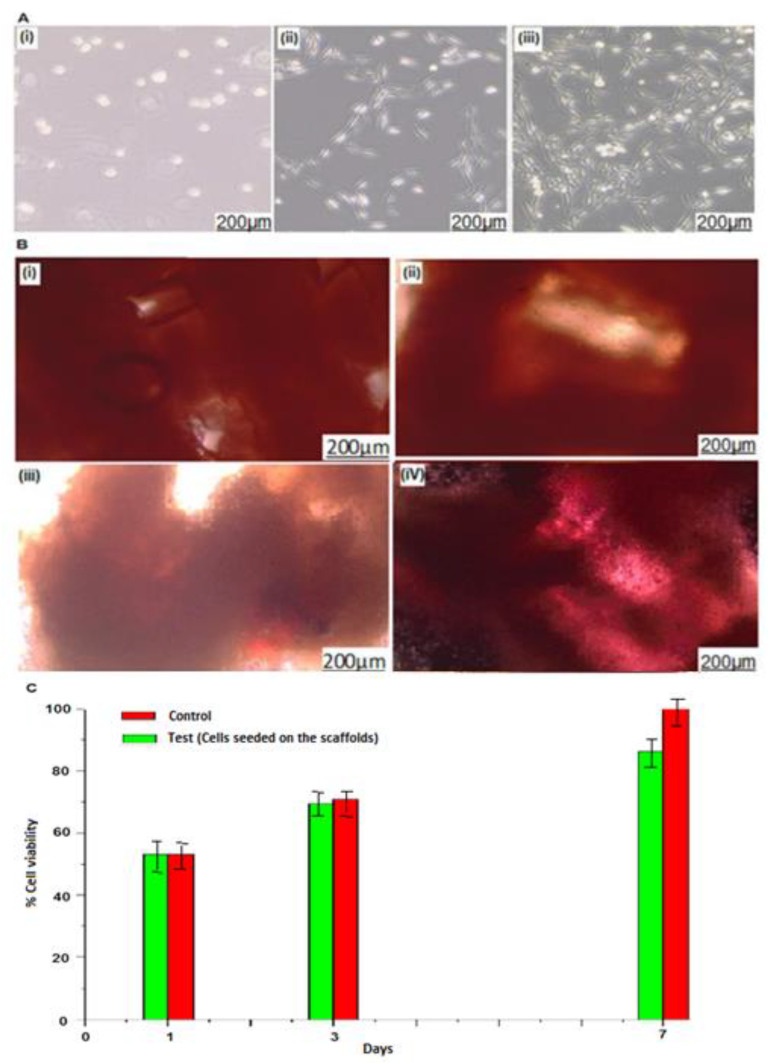
Images for (**A**) assessment of osteoblast-like MG63 cell differentiation on different days, where (**i**) depicts day zero, (**ii**) depicts day 2, and (**iii**) depicts day 3, (**B**) cell–scaffolds in different time intervals, where (**i**) depicts day zero (scaffold without cells as reference), (**ii**) depicts day 1 (cell-scaffold), (**iii**) depicts day 3 (cell-Scaffold) and (**iv**) depicts day 7 (cell-scaffold), and (**C**) cell viability performed on the 3D cell–scaffold within seven days using light microscopy (LC micro image analysis software).

Osteoblast-like MG63 cells were cultured successfully as depicted in Figure 3A, where Figure 3(Ai) depicts the initial culturing stage (day 0) of osteoblast-like MG63 cells which appeared to be round-like in shape when observed under light microscope. Figure 3(Aii) depicts day 2 of the osteoblast-like MG63 cell growth; it was observed that the cells changed from being round-like in shape and adapted to a flat-like shape as they differentiated. The osteoblast-like MG63 cells were allowed to differentiate until they reached approximately 70% to 90% confluence as depicted in Figure 3(Aiii). Furthermore, cell subculturing was carried out and subcultured cells were stored (−80 °C) for future use.

Proceeding from cell subculturing, the osteoblast-like MG63 cells were successfully seeded onto the novel 3D-printed biomaterial scaffolds. The cell–scaffold investigations carried out in this study were cell adhesion, cell proliferation, and cell viability. The 3D-printed biomaterial scaffolds were imaged for one, three, and seven days. An optical microscope was used to image the potential abilities of cell–scaffold adhesion, confluence, and proliferation. Figure 3(Bi) depicts day zero of the 3D-printed biomaterial scaffold without osteoblast-like MG63 cells, while Figure 3(Bii) depicts day 1 (24 h) of the 3D-printed biomaterial scaffold seeded with osteoblast-like MG63 cells; it was observed that the 3D-printed biomaterial scaffold seeded with osteoblast-like MG63 cells had approximately 5–10% confluence of osteoblast-like MG63 cells on its surface for day 1 when compared to the surface of the 3D-printed biomaterial scaffold (control) in day zero. Figure 3(Biii) depicts day 3 of the 3D-printed biomaterial scaffold seeded with osteoblast-like MG63 cells, and it shows 60–70% confluence of osteoblast-like MG63 cells on the 3D-printed biomaterial scaffold surface, while Figure 3(Biv), which depicts day 7, shows approximately 90–100% confluence, nearly covering the entire surface of the 3D-printed biomaterial scaffold. Complementing these results, it was also observed that after day one, cells had adhered and grown onto the 3D-printed biomaterial scaffold (Figure 3(Bii)). Figure 3(Biii) shows the osteoblast-like MG63 cells starting to form colonies on day 3, and it was also observed that the colonies were larger and denser on day 7 (Figure 3(Biv)), confirming the potential of cell growth, proliferation, and differentiation onto the 3D-printed biomaterial scaffold.

The novel 3D-printed biomaterial scaffold’s cell viability was assessed using an MTS cytotoxicity assay, and the results are depicted in Figure 3C. From the obtained analyzed results, it was observed that the novel 3D-printed biomaterial scaffolds were biocompatible with osteoblast-like MG63 cells. Hence, Figure 3C in the investigation of the osteoblast-like MG63 cells on the 3D-printed biomaterial scaffolds reveals that the growth rate of the osteoblast-like MG63 cells in the two groups (the control and cell scaffolds) was not significantly different (*p* > 0.05). Therefore, this result validated the biocompatibility of the 3D-printed biomaterial scaffold, showing that the osteoblast-like MG63 cells are viable in the 3D-printed biomaterial scaffold over seven days. It was also observed in both groups (control and cell scaffolds) that each day, the cells increased in number compared to the previous days. This reveals/confirms that the cells proliferate within the scaffold and differentiate over time.

**Figure 4:**
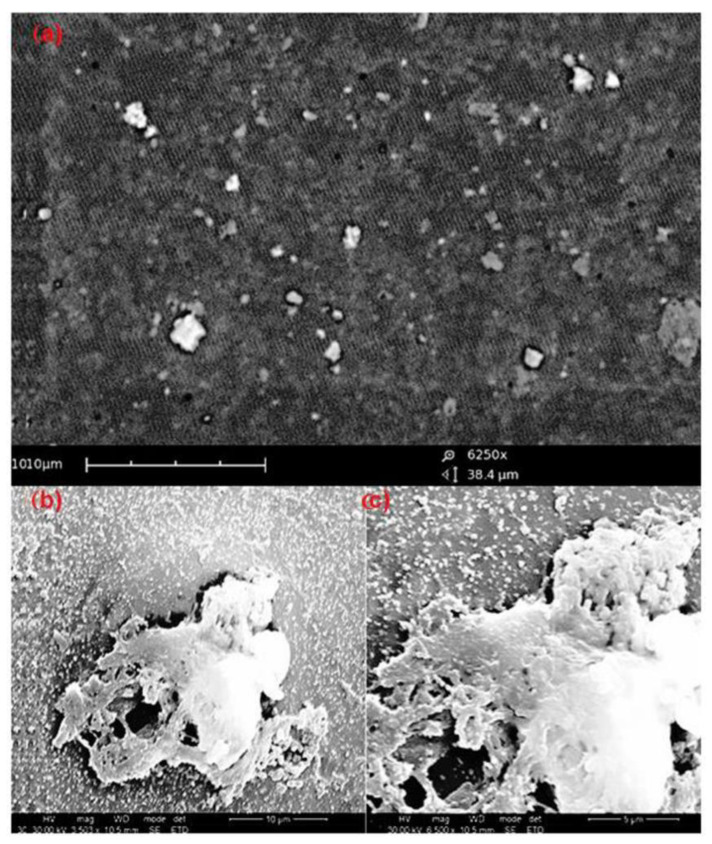
The SEM pictures of osteoblast-like MG63 cells adhered to the 3D-printed biomaterial scaffold on day seven, whereby (**a**) depicts reference scaffold (scaffold without cells), (**b**) depicts day 7 cell adhered on the surface of the printed scaffold, (**c**) depicts magnified day 7 of cell adhered to the surface of the printed scaffold.

Supporting the above results, scanning electron microscopy (SEM), Figure 4, was used to confirm cell attachment/cell adhesion onto the 3D-printed biomaterial scaffold. However, observing cell attachment/cell adhesion is not an easy process and is affected by many aspects such as environmental factors, material surface properties, and cell behavior [52]. One problem that was encountered during sample preparation was the issue of marking the samples. During sample preparation, it was observed that marking the sample was difficult to achieve. This led to difficulties in identifying the cells seeded on the sides of the scaffold. The determination of the correct side to perform the SEM analysis arose, but through trial and error, the cells were identified, as observed in Figure 4. The SEM images on the 3D-printed biomaterial scaffolds seeded with osteoblast-like MG63 cells and the 3D-printed biomaterial scaffold without cells (control) are depicted in Figure 4. Figure 4a depicts the 3D-printed biomaterial scaffold without cells (control) subjected to similar conditions as the 3D-printed biomaterial scaffold seeded with osteoblast-like cells (Figure 4b,c). Figure 4b shows cells attached to the surface of the 3D-printed biomaterial scaffold compared to the control scaffold (Figure 4a). Therefore, cell adhesion on the rough surface is highly advantageous for the development of implanted devices.

**Figure 5:**
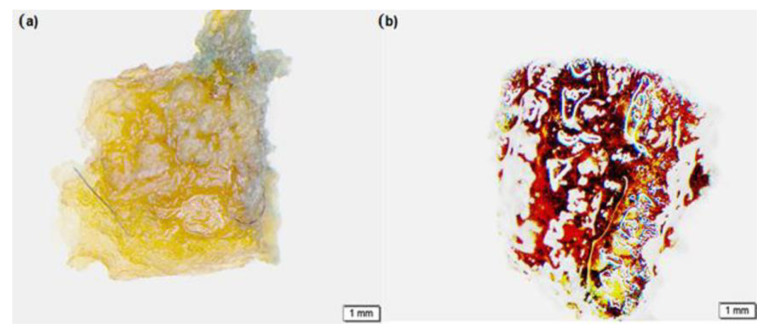
Images from Leica microsystem (Leica MZ10 F): (**a**) the scaffold without cells (control) and (**b**) the scaffold with cells, both treated with methylene blue.

Complementing the above results, a Leica microsystem (Leica MZ10 F) was used to confirm cell distribution and cell proliferation along the surface and inner pores of the 3D-printed biomaterial scaffold treated with methylene blue. The need for marking the sample side of the 3D-printed biomaterial scaffold was solved using this approach. With the Olympus software, it was possible to rescan the images and observe a more detailed distribution of cells along the scaffold surface. The nuclei appeared stained red and the matric appeared yellowish allowing a more accurate observation. Figure 5b represents day 7 of the scaffold seeded with cells, substantiating the previous data presentation, showing cells spreading along the scaffold, while Figure 5a represents the scaffold without cells (the control) subjected to a similar condition as the scaffold seeded with cells.

### 2.5. The In Vitro Studies of Human BMP-7 in the 3D-Printed Biomaterial Scaffolds

This study evaluated how the protein/growth factor (e.g., BMP-7) was released in vitro from the novel developed 3D-printed biomaterial scaffold. The purpose of the in vitro experiments was to assess how well the 3D-printed biomaterial scaffold might perform in vivo and to determine whether the BMP-7 was loaded/attached and can be released from the printed biomaterial scaffold in a way that resembled the natural healing process of bone fractures. Therefore, the 3D-printed biomaterial scaffold was loaded with 40.45 ng/mL of the BMP-7 and the in vitro release kinetics of the protein were measured.

**Figure 6:**
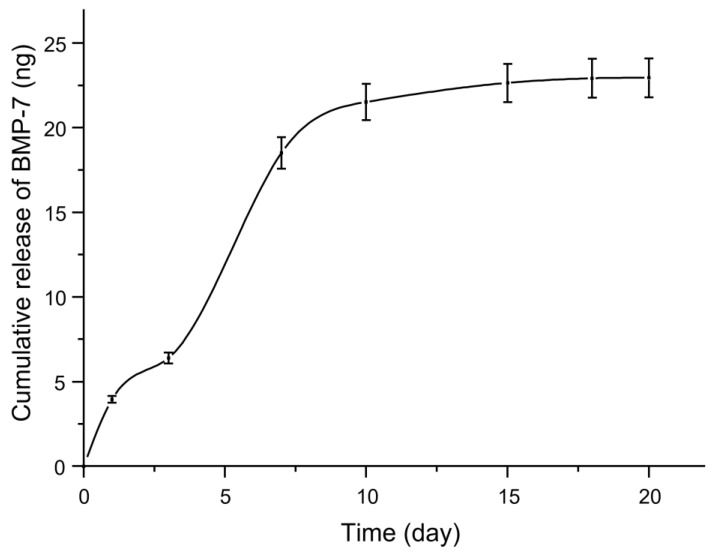
Depicts the in vitro release profile of BMP-7 from a 3D-printed biomaterial scaffold, with each data point representing the mean (*n* = 3).

Figure 6 shows the cumulative in vitro release profile of BMP-7 with the supernatant analyzed employing a human BMP-7 ELISA Kit. Hence, Figure 6 depicts two phases of in vitro BMP-7 release from the 3D-printed biomaterial scaffolds: an exponential release phase observed from day 1 to approximately day 10, followed by a plateau from day 11 up to the last day of the experiment. This in vitro release profile of BMP-7 from the scaffold is characteristic of an initial burst release followed by a sustained release, which has been shown to promote bone regeneration more effectively than sustained release alone, as reported previously [53,54]. In the 3D-printed biomaterial scaffold, BMP-7 was proposed to adhere to the biomaterial scaffold’s surface (since PEI has strong interaction with proteins), resulting in the early burst release. However, maintaining a steady release of BMP-7 is also important, as high concentrations could cause adverse side effects such as heterotopic bone formation, edema, or inflammatory reactions.

Cell viability was evaluated using an MTS cytotoxicity assay, with results consistent with those presented in Section 2.4, Figure 3C. The investigation focused on osteoblast-like MG63 cells growing on 3D-printed biomaterial scaffolds containing BMP-7, and it was observed that no significant difference (*p* > 0.05) in the growth rate of MG63 cells between the control group and the cell scaffolds with proteins. This finding indicates that the 3D-printed biomaterial scaffold is biocompatible, as the MG63 cells remained viable in the scaffold for seven days. Moreover, both the control group and the cell scaffolds demonstrated an increase in the number of cells each day compared to the previous days.

### 2.6. Clinical and Histological Analysis

There was no postoperative complication or adverse reaction such as abnormal bleeding during the healing of all surgical sites. There was a minimal sign of inflammation (e.g., swelling), and after 3–6 days postoperatively, the rabbits moved without notable pain/limitation. The 3D-printed biomaterial scaffolds were intact within the defects at the time of termination and collection of samples. Healing progressed smoothly without major incidents in the rabbits, and no postoperative complications were noted during the 8-week study period.

#### 2.6.1. Histological Examination for Group One (Week One)

**Figure 7:**
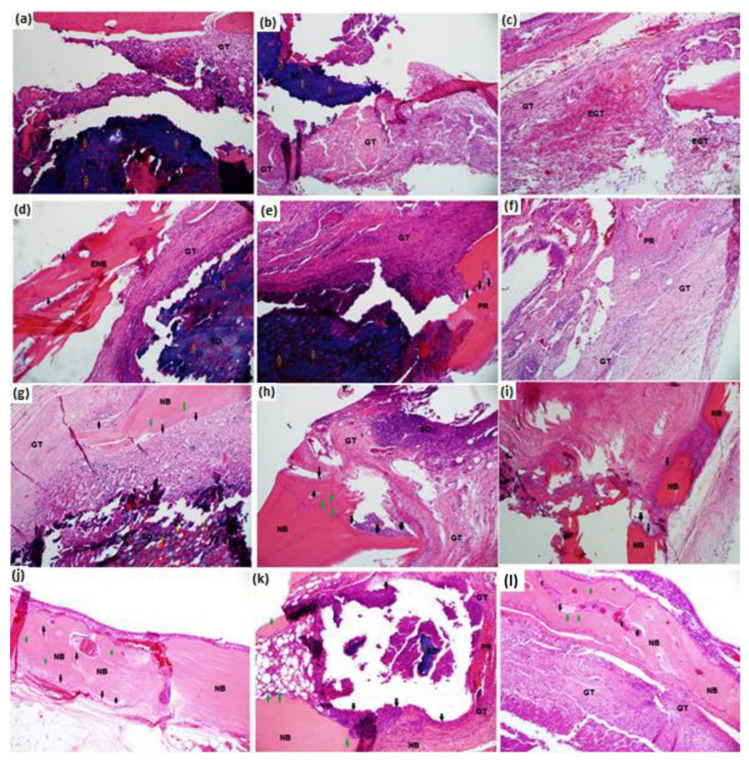
Histological images of (**a**) scaffold with BMP-7, (**b**) scaffold without BMP-7, (**c**) an empty nasal defect at week one postoperative and histological images of (**d**) scaffold with BMP-7, (**e**) scaffold without BMP-7, (**f**) an empty nasal defect at week two postoperative, and histological images of (**g**) scaffold with BMP-7, (**h**) scaffold without BMP-7, (**i**) an empty nasal defect at week 4 postoperative and histological images of the (**j**) scaffold with BMP-7, (**k**) scaffold without BMP-7, (**l**) an empty nasal defect at week 8 postoperative, with H&E staining (×10 magnification): GT = granulation tissue; SD = scaffold mostly represented by the space; yellow arrows = blood vessels within the scaffold, ENB = early new bone development, Black arrows = osteoblast, PR = periosteal reactions, NB = new bone development, Green arrows = osteocytes, Green star = bony spicules.

Figure 7 depicts photographic images of the embedded transverse sections of the scaffold with BMP-7 (protein), scaffold without BMP-7, and the defect without any scaffold (control), respectively, (Figure 7a–c) at week 1 postoperative histology.

A reported histological result for Figure 7a (scaffold with BMP-7) reveals a visible, large induced nasal defect and was filled with mixed eosinophilic and basophilic material (the biomaterial scaffold) which was surrounded by inflammation reaction and granulation tissue. Inflammation appeared mixed with macrophages and heterophils (possibly for fighting against pathogens and apoptotic cells) predominating and a few macrophages containing granular basophilic material. Necrotic bone fragments are present in the granulation tissue with surrounding macrophages (necrotic bone fragments could have resulted from the pieces of bone remains during the induction of the nasal bone defect). In Figure 7b (scaffold without BMP-7), a large defect in the nasal bone is visible, which was filled with granulation tissue as well as spicular to amorphous and a mix of eosinophilic and basophilic material (the biomaterial scaffold). Necrotic bone was prominent, and there was a marked inflammatory reaction with numerous degenerate heterophils. In Figure 7c (an empty defect without any biomaterial scaffold), there is a large visible defect in the nasal bone, which was filled with hemorrhage and fibrin, but there are areas with necrotic bone fragments and surrounding inflammation which includes macrophages and multinucleated giant cells. Early granulation tissue formation was noted as well. Therefore, all the defects in week one indicated no osteoblastic activities and a negligible amount of bone formation.

#### 2.6.2. Histological Examination for Group Two (Week Two)

Figure 7 depicts photographic images of the embedded transverse sections of the scaffold with BMP-7, scaffold without BMP-7, and the empty defect (control), respectively (Figure 7d–f), at week two postoperatively. As reported in the histological results in Figure 7d (scaffold with BMP-7), a focal defect in the nasal bone was noted, which was filled primarily by similar amorphous to spicular and a mix of eosinophilic and basophilic material (the biomaterial scaffold). There was surrounding inflammation consisting of mixed macrophages and heterophils with fibrosis and granulation tissue development. Early new bone development with osteoblast activity was noted as well. In Figure 7e (scaffold without BMP-7), the defect in the nasal bone is still visible, and is filled with a mix of eosinophilic and basophilic material (the biomaterial scaffold), with inflammation consisting of macrophages, some of which contain phagocytized basophilic granular material. There was mild early surrounding fibroplasia, but spicules of necrotic bone with surrounding macrophages were noted as well. A few heterophils and scattered lymphocyte and plasma cells were noted as well. Moderate periosteal reactions with active osteoblasts were noted on the edge of the bone defect. Figure 7f (an empty defect) shows a visible defect that was filled with granulation tissue and clusters of lymphocytes and plasma cells with a few interspersed adipocytes. Moderate hemorrhages and fibrin deposition were also noted. The edge of the defect shows mild osteoblastic activity, and there is a marked periosteal reaction. Focally, there was a fragment of necrotic bone with surrounding macrophages and multinucleated cells. Therefore, all the defects in week two indicated osteoblastic activities; though in Figure 7d (biomaterial scaffold with BMP-7), early bone formation was thought to be more prominent compared to the other defects.

#### 2.6.3. Histological Examination for Group Three (Week 4)

Figure 7 depicts photographic images of the embedded transverse sections of the scaffold with BMP-7, scaffold without BMP-7, and the empty defect (control), respectively (Figure 7g–i), at week 4 postoperatively. As reported in the histological results in Figure 7g (biomaterial scaffold with BMP-7), a defect was visible in the nasal bone, which was filled with granulation tissue and a mix of eosinophilic and basophilic material (the biomaterial scaffold). There was a surrounding inflammation lesion consisting of heterophils and macrophages. A few of the macrophages contained granular basophilic material. New bone formation with prominent osteoblastic activity was noted between the edge of the defect and the foreign material (the biomaterial scaffold). In Figure 7h (scaffold without BMP-7), a segment of the nasal bone was interrupted and filled with large quantities of granulation tissue and inflammation. New bone formation was prominently visible between the defect and the granulation tissue with marked osteoblastic activity. In Figure 7i (an empty defect), the lesion was visible, but this one was very subtle and consisted of a focal segment of bone thinning and new bone formation. This new bone showed increased osteocyte density, and there was osteoblastic activity on the periosteal surface with the production of thin bone spicules.

#### 2.6.4. Histological Examination for Group 4 (Week 8)

Figure 7 depicts photographic images of the embedded transverse sections of the scaffold with BMP-7, scaffold without BMP-7, and the empty defect (control), respectively (Figure 7j–l) at week 8 postoperatively. As reported in the histological result in Figure 7j (scaffold with BMP-7), the nasal bone was complete (with bone formation); however, there was an area showing thinning and depression in the sinus space. This was associated with a focus of inflammation in the adjacent subcutis consisting primarily of macrophages and heterophils, which was associated with foreign material of the mix (combination) of basophilic and eosinophilic types. There was marked surrounding fibrosis. The adjacent thin bone segment was new with an increased number of osteocytes as well as osteoblast activity. In Figure 7k (scaffold without BMP-7), there was a marked thickening of the bone with the production of mature bony spicules and medullary cavities containing adipocytes, and this was bordered by a thinner segment. Nevertheless, there was prominent new bone formation with an increased osteocyte density. This section was associated with a moderate periosteal reaction, and there were small medullary cavities containing prominent lining osteoblasts. However, a small defect was still visible, but this was filled with fibrous connective tissue and fibroblasts, though osteoblastic activity was still present on the edges. In Figure 7l (an empty defect), a small segment with thin new bone formation was noted, showing increased osteocyte density and osteoblastic activity on the periosteal surfaces and perivascular spaces.

## 3. Discussion

The traditional way to repair or treat non-union (critical-sized) bone defects has been focused on the use of autografts or orthopedic implants, and allografts. However, these techniques have limitations, such as inadequate biomechanical properties and the lack of available donors [55,56,57]. More recently, the employment of biomaterial scaffold for bone tissue engineering has emerged as an alternative technique to overcome the limitations of the mentioned approaches [56,58,59]. Biomaterials used to prepare scaffolds for tissue engineering applications must meet certain requirements, these include printability (for 3D-printed scaffolds), biodegradability, and biocompatibility, amongst others. Alginate is widely used in tissue engineering applications as a biomaterial for scaffold fabrication since it is a biocompatible natural polymer, containing two monosaccharide units: mannuronic acid and guluronic acid. Alginate is also widely utilized as a protein (growth factors) delivery system, for cell encapsulation, and drug delivery systems [60,61,62,63,64]. A stable hydrogel can be formed when alginate is mixed with divalent/trivalent cation live Ca^2+^. There is an ionic interaction between the cations and the negatively charged carboxyl groups of the guluronic acid units of the alginate chain. Hence, gelation is based on non-covalent interaction [65,66]. Therefore, biomaterial scaffolds made of alginate present great potential to be used in bone tissue engineering applications, even though it has low compressive strength and modulus and it lacks bioactivity; nevertheless, it can deliver living cells and growth factors/protein [67,68].

Polyethyleneimine (PEI) is a branched polycationic polymer that contains a high density of ionizable tertiary, secondary, and primary amino groups and has a strong interaction with proteins. PEI has been employed widely in tissue engineering and promotes cell growth. PEI has also been utilized to enhance protein loading on solid surfaces and PEI has been introduced onto the surfaces of organic or inorganic solid spheres using techniques such as covalent grafting, spray drying, electrostatic adsorption, and layer-by-layer assembly. Murakami et al. [69] produced PEI-coated hydroxyapatite with various fractions of PEI via a spray-drying technique. Furthermore, Xia et al. [70] employed a layer-by-layer technique to produce chemically crosslinked PEI using glutaraldehyde. Therefore, PEI can form a polyelectrolyte (PEC) layer following electrostatic adsorption on a negative substrate [71], such as alginate, to create a complex with potentially enhanced capabilities. Silica gel powder is an exemplary inorganic filler component commonly included in bone scaffolds. Bioactive materials containing silicon species have demonstrated enhancement of osteogenesis. Silica could, thus, be included in the bioink for improved bone tissue biosimulation.

The bioactivity and mechanical strength of alginate-based scaffolds can be improved by modifying alginate through the introduction or reinforcement of inorganic materials such as hydroxyapatite or silica gel, amongst others. Traditional reinforcements of inorganic fillers are based mainly on the physical interactions with the matrix hydrogel in which van der Waals/secondary forces such as hydrogen bonding, dipolar interactions, and London dispersion forces are involved [72]. These interactions enhance the hydrogel properties since they generate strong adhesion between the hydrogel matrix and the reinforced inorganic fillers [73]. In our lab, a 3D-printed biomaterial scaffold containing alginate plus silica gel powder (as inorganic filler) was printed in a polyethyleneimine (PEI) solution to form a polyelectrolyte complex (PEC) (3D Alg-PEI/Si). The 3D-printed Alg-PEI/Si scaffold was a promising biomaterial hybrid composite because of its confirmed outstanding potential biocompatibility, porous architecture, and acceptable mechanical strength acting as an artificial extracellular matrix necessary for specific bone tissue engineering applications. The objectives of this study were to evaluate the 3D-printed Alg-PEI/Si scaffold construct’s potential in regenerating bone defects and determining the effect of the added BMP-7 components in vivo. The rabbit models were previously shown to be appropriate for evaluating bone substitutes at the early stages of healing. Furthermore, previous studies suggested that 5 mm diameter defects are a sufficient size for rabbit nasal bone to observe the bone regeneration progress. In the rabbit model, the dimension and location of the defect were considered effective [74]. The same size 3D-printed biomaterial scaffolds were implanted in all groups except in the control group to avail the same amount of space for bone regeneration.

Histological results revealed from specimens harvested in week one that almost all of the defects showed no signs of osteoblastic activities from the rabbits terminated in week one. However, healing and osteoblastic activities started unevenly in different defects in the form of new bone growth in the margins of the defects as well as in the form of a small island of woven bone for some of the defects in animals terminated in week two. As the weeks progressed, there were bonier islands observed compared with the histological observations of the previous weeks. Part of the newly formed bone had an appearance of lamellar structure. The fibroconnective tissue filled the porous structure of the scaffold, and we occasionally observed the ingrowth of the maturing bone to the scaffold structures starting from the surface of the nasal bone where the scaffold was fixed. Dense fibrous connective tissue surrounds these bony islands. Connective tissue ingrowth into the pores of the scaffold and the mineralization process was observed. The formation of bone seemed to progress gradually inside the scaffolds from the dense fibro connective tissue to the newly formed bone through the mineralization of the tissue. There was an observed inflammation reaction with foreign body giant cells, lymphocytes, and macrophages. As healing progressed, the inflammation reaction was minimal and mainly fibrous connective tissue was observed inside the scaffold. The inflammatory response was strong in the side of the scaffold facing the periosteum and skin. Therefore, foreign-body-type inflammation reactions composed of giant cells, macrophages, granulocytes, and lymphocytes as well as plasma cells were mostly found inside the upper part of the scaffold. Despite the presence of inflammatory reactions, the fibrous connective tissue and bone formation were present.

In this study, the assessment of the bone regeneration process was carried out using two different implants (the scaffold with BMP-7 and the scaffold without BMP-7), and we compared the bone regenerative potential of the studied materials histologically using rabbits as the animal experimental model. Both defects with scaffolds appeared to become progressively smaller with time. However, defects containing the biomaterial scaffold that had BMP-7 were observed to heal to near completion in week 8 compared to the defect with scaffold without growth factors and the empty defect. There was evidence of the scaffold being resorbed and the scaffold appeared to make a significant difference in the healing time required. Furthermore, the histological observation in week 8, Figure 7k, show a different change that may need further studies; however, for the purpose of this research, the results have shown enough information to indicate the potential abilities of the novel 3D-printed scaffold for bone tissue regeneration.

## 4. Materials and Methods

### 4.1. Materials

Osteoblast-like MG63 cells were purchased from PromoLab (Pty) T/A Separations (Johannesburg, South Africa) and the MTS cell Proliferation Colorimetric Assay Kit was purchased from Whitehead Scientific (Pty) Ltd. (Cape Town, South Africa). Alpha minimum essential medium (α-MEM) (Gibco), fetal bovine serum (FBS; Biochrom, Berlin, Germany), DAPI, methylene blue, amphotericin B solution, gentamicin solution, trypsin-EDTA solution, RNAse, triton X-100, poly (ethyleneimine) solution (PEI, 50% *w*/*v* in water, M_w_ = 750,000), sodium alginate (NaAlg, Product number: W201502, sodium alginate), silica gel powder (Si, M_w_ = 60,000), hydroxyapatite (HA, M_w_ = 502.31), nanoclay surface modified (Nanoclay, contains = 0.5–5 wt.% aminopropyltriethoxysilane and 15–35 wt.% octadecylamine), formaldehyde (M_w_ = 30.03), phosphate buffer saline, Dulbecco’s modified eagle’s medium (DMEM), vectashield^®^, glutaraldehyde solution (M_w_ = 100.116), ethanol(M_w_ = 46.07), Bone Morphogenetic Protein 7 (BMP-7) Human (Osteogenic protein 1), and alexafluor-conjugate (Anti-Actin Antibody, Alexa fluor^®^ 488 conjugated, clone C4) were purchased from Sigma-Aldrich (St. Louis, MO, USA). Ketamine HCL (dissociative anesthetic), xylazine HCL (sedation, anesthesia, muscle relaxation, and analgesia), sodium phenobarbitone (M_w_ = 254.22), lidocaine (M_w_ = 270.80), antisedan (M_w_ = 212.29), mobic (M_w_ = 352.4), and temgesic^®^ (M_w_ = 467.6) were purchased from Dev Life corporation. All other reagents/substances were of standard analytical grade and were utilized as procured.

### 4.2. Preparation of the Novel In Situ 3D-Printed Biomaterial Scaffolds from Different Inorganic Fillers

The 3D-printed biomaterial scaffolds were fabricated using sodium alginate (NaAlg) impregnated/reinforced with inorganic fillers [silica gel powder (Si), nanoclay, and hydroxyapatite (HAP)], and printed into a PEI solution, as previously reported elsewhere [35]. Briefly, Alg-PEI/Si, Alg-PEI/HAP, and Alg-PEI/nanoclay 3D-printed biomaterial scaffolds were engineered as follows: Ultra-filtrated purified deionized water (Millipore, France) was used in the preparation of the biomaterial ink, by hydrating sodium alginate (200 mg/mL). A preprepared inorganic filler [Si (200 mg/mL) or HAP (200 mg/mL) or nanoclay (200 mg/mL)] dispersion was then combined with the alginate solution. Poly (ethyleneimine) solution (PEI) was prepared separately by adding 40 mL of the as received 50% *w*/*v* PEI aqueous solution to 100 mL ethanol to make the “forming” medium.

**Figure 8:**
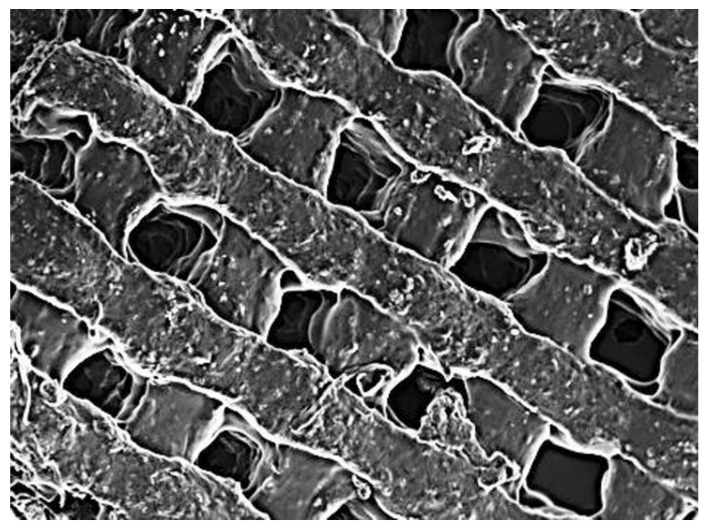
The examples of the physical photo of the 3D-printed biomaterial scaffold.

The 3D-printed biomaterial scaffold (Figure 8) was engineered using 3D Bioplotter^®^ (EnvisionTEC, GmBH, Gladbeck, Germany). For the desired structure (3D biomaterial scaffold) to be printed, the 3D printer uses a computer-aided design (CAD). Two moduli are contained in the control of the 3D Bioplotter^®^ and the software of STL, which involves (i) the 3D Bioplotter^®^ software for STL data importation and structure (e.g., 3D biomaterial scaffold) fabrication and (ii) the visual machine software for material parameters and machine control. The 3D biomaterial scaffolds were printed at a temperature of 37 °C and a 30 cc cartridge was used to feed the hydrogel-inks. A plastic needle (nozzle diameter 0.41 mm) was used to dispense the hydrogel-inks at a deposition speed of 10.1 mm/s and pressure of 0.8 Bar into the petri dish containing PEI dissolved in ethanol (as the solidifying solution). The layers were deposited at an angle of 0 and 90° per twofold layer into the underlying layer in a solidifying solution containing PEI. The average solidification time per layer was noted as the layers were successively printed. Table 2 describes the modified parameters used to print the scaffolds. The 3D-printed scaffolds (Figure 8) were washed thrice with double deionized water/ethanol and then dried at room temperature (25 °C) to a constant weight.

### 4.3. Determination of the Biomaterial’s Complexation and Gelation Kinetics

The complexation and gelation kinetics of the precursor hydrogels (Alg/Si and Alg) and the composite hybrid (Alg-PEI/Si) were assessed using ElastoSens^TM^ Bio^2^ (Rheolution instruments, Montreal, Canada). Briefly, a small amount of approximately 3 mL of hydrogel (200 mg/mL) in its initial state (using printing needles) was poured into a disposable, cylindrical sample holder with a flexible bottom membrane. For complexation analysis, the hybrid composite (Alg-PEI/Si) was prepared by adding the hydrogel (Alg/Si) into a poly(ethyleneimine) solution (40 mL PEI of 100 mL ethanol) that was already in the sample holder. The gelation kinetic (ionic interaction), complexation, and viscoelastic (G′ and G*) properties of the composite Alg-PEI/Si hybrid were then determined over time. The ElastoSens^TM^ Bio^2^ technique processes the shear storage (G′) and loss (G″) moduli of gels (hydrogels) as a function of temperature/time. The gentle mechanical vibration of the gel (hydrogel) confined in the sample holder provides a response that was detected via laser for analysis. The appropriate time it may take to print the next layer without any deviations and the ionic interaction between the two ionic biomaterials was determined.

### 4.4. Determination of Biomechanical Properties of the 3D-Printed Biomaterial Scaffolds

The biaxial tensile strain within the composite 3D-printed biomaterial scaffold was tested per an ASTM standard protocol (D882-02), utilizing a BioTester 5000 (CellScale, Waterloo, ON, Canada) equipped with 5 N load cells (*n* = 3). Following the scaffold’s equilibration, the 3D-printed biomaterial scaffold samples were suspended in PBS (pH 7.4; 37 °C) and attached to a stainless-steel clamping mechanism to evaluate the scaffold rigidity, strength, and displacement. The BioTester was balanced, calibrated, and moved apart for 3200 mm before mounting the scaffold. The 3D-printed biomaterial scaffolds were subjected to a preload of 20 mN once mounted, and thereafter, 5 N of strain was applied as ramp force until displacement had plateaued to simulate prolonged strain.

### 4.5. Morphological and Pore Size Evaluation of the 3D-Printed Biomaterial Scaffold

Scanning electron microscopy (SEM) FEI Noval NanoLab SEM (FEI Company, Hillsboro, OR, USA) was utilized to examine the surface morphology (pore interconnectivity and roughness) of the 3D-printed biomaterial scaffold. Briefly, the 3D-printed biomaterial scaffold samples were cut into 2 mm-thick discs (perpendicular to the long axis of the cylinder) before SEM analysis. The cut sections were sputter-coated using the gold isotope. These scaffolds’ section samples were mounted using the aluminum spud with an EPI coater (SPI Model sputter-coater and control unit, Hester, PA, USA). The coating of the samples took 60 s while subjected to constant nitrogen gas conditions; then, the samples were examined employing an FEI Nova NanoLab SEM (FEI Company, Hillsboro, OR, USA) [35]. The pore size dimensions were measured employing ImageJ software. The manual mode of the ImageJ analyzer was used for the measurement of the average diameter of the pores. At least 40 pores were assessed at each of five SEM micrographs for each scaffold type. Randomly selected pores were analyzed for both long and short pore axis [75].

### 4.6. Porositometric Evaluation of the 3D-Printed Biomaterial Scaffold

The 3D-printed biomaterial scaffold porosity was calculated from the microscopic images of the scaffold following the theoretical approach of Landers et al. [76] as shown in Equation (1):

(1) $$P=\frac{V_{scaffolds}}{V_{cube}}=1-\frac{\pi }{4}\cdot \frac{{\left(d\right)}^{2}}{d_{1}d_{2}}$$

where

*P* = scaffold porosity;

*d* = scaffold fiber diameter;

*d*_1_ = scaffold fiber spacing and *d*_2_ = layer thickness within each different structure [77].

### 4.7. Biomineralization Evaluation of the 3D-Printed Biomaterial Scaffold

The 3D-printed biomaterial scaffold’s biomineralization investigation was performed using simulated body fluid (SBF, pH 7.4, 37 °C). The immersion solution was prepared using NaHCO_3_ (4.2 mmol/L), CaCl_2_ (2.6 mmol/L), NaCl (14 mmol/L), MgCl_2_·6H_2_O (1.6 mmol/L), Na_2_SO_4_ (0.5 mmol/L), K_2_HPO_4_·3H_2_O (1 mmol/L), and KCl (3.0 mmol/L) in deionized water. The solution was adjusted to a pH of 7.4 using tris-hydroxymethyl aminomethane ((CH_2_OH)_3_CNH_2_) and 1 M HCl at 37 °C. The samples were immersed in simulated body fluid (SBF) in a plastic bottle and placed in an orbital shaker (YIHDER LM-530, YIHDER Co., Ltd., Taipei, Taiwan) (120 rpm) at 37 °C. After 7 days of immersion, the samples were taken out of the solution and dried completely for 24 h [78]. The dried samples were taken for FTIR and SEM analysis to do the EDX elements analysis.

### 4.8. The In Vitro Assessment of the Osteoblast-like Cells

The cell viability of the developed novel 3D-printed biomaterial scaffold was evaluated using an MTS assay. The standard culture medium was used as a control medium in this investigation. The MTS assay aimed at evaluating the cell viability of the novel 3D-printed biomaterial scaffold with osteoblast-like cells before implanting the scaffold in a living organism. Therefore, the objective of the MTS assay test was to determine whether the cells were metabolically active on the 3D-printed biomaterial scaffolds [41,79].

#### 4.8.1. Cell Culturing Method for the Osteoblast-like Cell Line

This study utilized the osteoblast-like MG63 cells that were derived from human osteosarcoma. The osteoblast-like MG63 cells were chosen because they express several characteristic features of osteoblasts, and they are amongst the best candidate for the evaluation of cell viability, cell adhesion, and cell proliferation in bone scaffold investigations. Briefly, osteoblast-like MG63 cells were grown as a monolayer in a prepared cell culturing medium made of alpha minimum essential medium (α-MEM) (Gibco) supplemented with 20% fetal bovine serum (FBS; Biochrom, Berlin, Germany), 1 mL of amphotericin B solution, and 1 mL of gentamicin. A 75 cm^2^ adhesive flask containing 15 mL of prepared cell culturing medium was used to culture the osteoblast-like MG63 cells at 37 °C in a humidified atmosphere containing 5% CO_2_ in the air. The cell culturing medium was changed every second day [40,80,81].

#### 4.8.2. Procedure for Seeding Osteoblast-like Cells into the Printed Biomaterial Scaffold

The 3D-printed biomaterial scaffolds (precut 14 mg/well scaffold, ca. 1 cm^2^ surface area) were sterilized by exposure to UV irradiation for 30 min, then washed with culture medium/PBS before placing them in 96-well plates [82]. Briefly, as described in Section 4.8.1, the osteoblast-like MG63 cells were allowed to grow as a monolayer until they reached 60% to 90% confluence. The cells were harvested using a trypsin-EDTA solution following the cell supplier’s protocol. The cells were centrifuged and resuspended in a prepared cell culture medium and counted using a hemocytometer. An aliquot (100 µL) containing 3 × 10^5^ cells was seeded on top of the novel 3D-printed biomaterial scaffold which was previously placed in 96-well plates. The culture medium of 100 µL was added to each well after two hours of seeding and then incubated in a humidified atmosphere at 37 °C containing 5% CO_2,_ with the medium changed every second day.

#### 4.8.3. Cell Viability Assay Assessment for the 3D-Printed Biomaterial Scaffold

The generally used cell viability testing method, the MTT assay, was substituted with an alternative MTS assay (Cell Titer 96 Ag Non-Radioactive Cell Assay, Promega Corporation, Madison, USA) in this research study to assess the cultured osteoblast-like MG63 cells on the 3D-printed biomaterial scaffold. The reason for not using the MTT cytotoxicity assay in this research study was because the reaction product of the MTT assay is water-insoluble formazan, which strongly attaches to the surface of the 3D biomaterial scaffolds and makes it impossible to obtain precise measurements. Hence, the MTS cytotoxicity assay was used because the formed reaction product is soluble in the cultured medium, which makes it possible to obtain precise measurements. Briefly, for different cell scaffolds cultured at time intervals, the medium was removed and replaced with 10% *v*/*v* of MTS solution in cultured medium to each well and incubated for 3 h. All absorbances were measured at a wavelength of 490 nm in a 96 multilabel reader Victor^TM^ X3 and the backgrounds of the well plates were measured at 690 nm. The results were presented as percentage relative cell viability (mean ± standard deviation) [41,79]. Equation (2) was utilized to calculate the percentage relative cell viability:

(2) $$\%Relative\;cell\;viability=\frac{OD\left(sample\right)-OD(blank)}{OD\left(control\right)-OD(blank)}\times 100$$

### 4.9. Osteoblast-like Cells Behavior in the 3D-Printed Biomaterial Scaffold

Techniques such as scanning electron microscopy (SEM), optical microscopy, and methylene blue staining were used to assess the proliferation and adhesion of osteoblast-like MG63 cells that were seeded onto the novel 3D-printed biomaterial scaffold.

#### 4.9.1. Evaluation of Cell Adhesion onto the 3D-Printed Biomaterial Scaffold

Cell adhesion was evaluated using optical microscopy and scanning electron microscopy (SEM) after culturing the cells for three or seven days. Briefly, the 3D-printed biomaterial scaffolds were cut into portions of equal size to determine if the cells were attached to the scaffolds. Phosphate buffered saline (PBS) solution was used to wash the scaffold and fixed it in a 1.5% *v*/*v* glutaraldehyde in PBS for 30 min. The 3D-printed biomaterial scaffolds were dehydrated by sequentially immersing in 50, 60, 70, 90, and 100% *v*/*v* ethanol series for 15 min each and dried in air. Finally, the samples were analyzed using SEM after being sputter coated with gold [39,40,41,83].

#### 4.9.2. Osteoblast-like Cell Staining Using Methylene Blue

The 3D-printed biomaterial scaffolds were cultured with cells for three or seven days and removed from the culture medium to be washed with phosphate-buffered saline (PBS) solution. Cell–scaffold samples were fixed with 1.5% *v*/*v* glutaraldehyde in PBS for 30 min. PBS was used to wash these samples twice, and then, they were immersed in methylene blue solution for 5 min. The samples were finally washed with PBS; then, cell adhesion and proliferation were evaluated using optical microscopy [40,81].

### 4.10. The In Vitro Studies of Protein (BMP-7) from 3D-Printed Biomaterial Scaffold

The 3D-printed biomaterial scaffolds were sterilized by exposing them to UV light for 24 h. The scaffolds were immersed in a BMP-7 solution (5 µg/mL) at 37 °C for the BMP-7 to attach to the surface of the printed scaffold, since PEI, which is part of the biomaterial scaffold, has a strong interaction with proteins. The unbound BMP-7 solution was measured to evaluate the BMP-7 loading rate of the 3D-printed biomaterial scaffold. The supernatant in the solution of the 3D-printed biomaterial scaffold was recovered by centrifugation at 500 rpm; then, the free-protein (BMP-7) concentration was determined using a human BMP-7 ELISA kit (ab282294).

The protein (BMP-7) released from the 3D-printed biomaterial scaffold was in vitro assessed using PBS (pH 7.4, 37 °C). Briefly, the 3D-printed biomaterial scaffold was cut to ten milligrams and incubated with 1 mL of phosphate buffered saline (PBS, pH 7.4) in 1.5 mL tubes at 37 °C in a rotary shaker at 45 rpm. Samples (supernatants) were collected at days 1, 3, 7, 10, 17, and 20. Then, 1 mL of supernatant was withdrawn at each time point and the 3D-printed biomaterial scaffolds were resuspended in 1 mL of fresh (PBS, pH 7.4). The amount of BMP-7 released at each time point was determined in the supernatant using a human BMP-7 ELISA Kit (ab282294).

### 4.11. The Design and Surgical Procedure for the In Vivo Intervention

**Figure 9:**
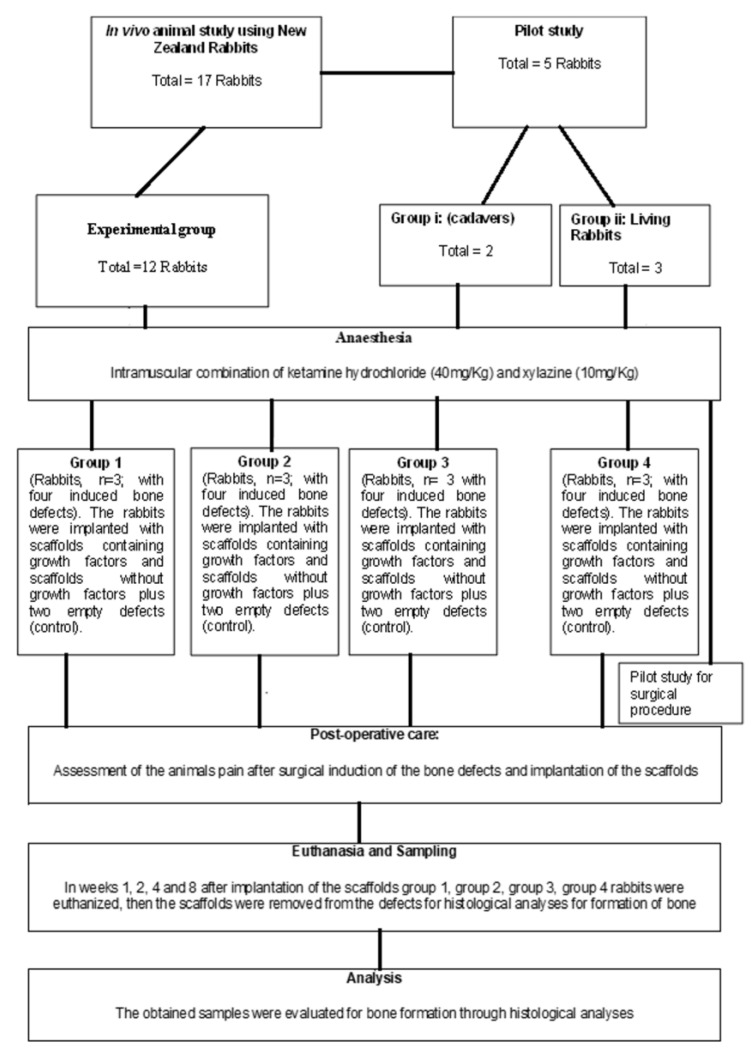
Schematic showing the in vivo studies model for the implanted scaffold.

The study used 17 New Zealand White Rabbits which were randomly assigned into groups, and 5 rabbits were assigned for a pilot study. The main study was conducted using 12 rabbits to observe bone healing over time. The study was designed to comprise four experimental groups (1, 2, 3, and 4), each with a sample size *n* = 3. The 3D-printed biomaterial scaffolds were evaluated in each group at different time intervals (1, 2, 4, and 8 weeks) after each rabbit’s termination. In the experimental groups, 3D-printed biomaterial scaffolds were utilized for the healing/regeneration of the induced critical-sized bone defects. Figure 9 depicts the schematic representation of the proposed experimental design for the in vivo studies.

**Figure 10:**
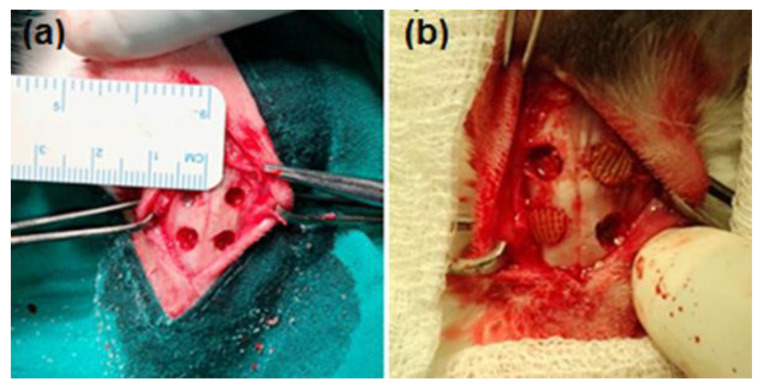
Schematic representation of the surgical procedure. (**a**) Four induced nasal critical-sized bone defects. (**b**) Example of how scaffolds were placed and the empty defects in the New Zealand White Rabbit.

The New Zealand White Rabbits were housed individually in cages before the surgical procedures. Then, the regeneration/formation of bone tissue in the induced critical-sized bone defects in rabbits was investigated. Each rabbit was induced with four bone defects as depicted in Figure 10a, where two defects were implanted with the 3D-printed biomaterial scaffolds (e.g., one defect contained a scaffold with BMP-7 and the other a scaffold without BMP-7) and the other two defects were empty as control (see Figure 10b for an example of how the scaffolds were placed and the empty defects). The rabbits were euthanized per group for a period of 1, 2, 4, and 8 weeks postoperative following implantation. Ethics approval was obtained for the use of the New Zealand White Rabbits from the Animal Ethics Screening Committee of the University of the Witwatersrand (Clearance Certificate No. 2017/10/64/D).

### 4.12. Surgical Procedure for the Intervention

Before the surgical intervention, the 3D-printed biomaterial scaffolds were sterilized using UV irradiation for 24 h. Following the sterilization process, the Dulbecco’s modified Eagle’s medium (DMEM), supplemented with 10% fetal bovine serum (FBS), was utilized to soak the 3D-printed biomaterial scaffold and incubated in an atmosphere of 5% CO_2_ at 37 °C (pH 7.4) for 24 h to equilibrate. The 3D-printed biomaterial scaffold was directly functionalized with a BMP-7 (also known as Osteogenic protein 1) using the physisorption strategy (also known as physical absorption). The physisorption strategy is the most straightforward strategy for embedding biomolecules in biomaterial scaffolds. Briefly, the preformed 3D-printed biomaterial scaffolds were immersed in a solution containing the BMP-7. The absorption of the BMP-7 was the result of electrostatic interaction [84]. Therefore, the BMP-7 solution was diluted to a concentration of 5 µg.mL^−1^ and then left to be absorbed on the 3D biomaterial scaffolds for 1 h before implantation [85]. The adsorption/attachment mechanism of BMP-7 onto the 3D-printed biomaterial scaffold surface was caused by the electrostatic attractive force between the binding groups of charged carboxylate in BMP-7 and the unreacted cation part of polyethyleneimine (PEI) in the 3D-printed biomaterial scaffolds [86,87,88].

All surgical procedures were performed in sterile conditions. The critical-sized bone defects were induced as previously described by Ishihara et al. [89] with minor modifications. Briefly, the rabbits were anesthetized using an intramuscular combination of ketamine hydrochloride (40 mg/Kg) and xylazine (10 mg/Kg). The anesthesia was maintained with isoflurane plus oxygen. The hair above the nasal bone was shaved and the skin was surgically prepared using F10 Skin Prep Solution; the F10 Skin Prep Solution is the skin ready-to-use disinfectant. It is a surgical spray that has a pre- and postoperative broad spectrum. This non-staining formula is easy to use, prevents the spreading of infections, and destroys micro-organisms; it does not irritate the skin and is bright in color, making it easily identifiable. Hence, it is highly beneficial in preventing postsurgical infections. Thereafter, 1.8 mL of 2% lidocaine containing 1:80,000 epinephrines was administered subcutaneously. Both the nasoincision suture line and nasal bone via perpendicular incision were exposed. A critical-sized nasal bone defect (5 mm in diameter) of approximately 2 mm thickness was induced (while preserving the nasal membrane) using a fissure bur with continuous saline irrigation. The 3D-printed biomaterial scaffolds were implanted into the defects. Following the creation of the critical-sized nasal bone defects and the implantation of the 3D-printed biomaterial scaffolds, the surgical site was sutured closed. To avoid nasal bone infections, the antibiotic baytril^®^ (enrofloxacin) was administered. The pain was managed with meloxicam [89,90].

### 4.13. Postoperative Care of the Rabbits

Postsurgery, the rabbits were closely monitored and observed for smooth recovery. The rabbits were maintained under a 12 h light: 12 h dark cycle while kept individually in separate cages at ±25 °C. They were fed per Wits Research Animal Facility’s (WRAF) feeding SOPs throughout the study. The rabbits maintained a good appetite for food and water for the study duration/length. The rabbits were weighed two times weekly during the period of the study. There was no necessity for supportive orthotic devices and no need for postoperative restriction on the rabbits’ activity. The rabbits were inspected twice daily for their welfare and other parameters for systemic complications such as the animal behavior, posture, weight, and feeding and drinking pattern. There were no morbidities or premature mortalities recorded as a result of the procedures.

### 4.14. Procedure for Euthanasia and Sample Collection

The rabbits were euthanized using IV sodium pentobarbitone (2 mL/Kg) via the ear vein. The euthanization happened at 1, 2, 4, and 8 weeks after the scaffold’s implantation. Then, implanted scaffold (the scaffold with the growth factors and the scaffold without the growth factors) and the empty defect samples were immediately harvested from the nasal bone of the rabbits. The removed specimens were stored in 10% buffered formalin until the histological procedures as described in Section 4.15.

### 4.15. Sample Preparation for Histological Examination

Following fixation of the specimens in 10% buffered formalin, the specimens were demineralized for 4 weeks using 14% EDTA. Representative specimen samples from the induced defects were trimmed according to Idexx standard operative procedure (IdexxSA-AP-26) and processed using routine histological tissue processing in an automated tissue processor with standard operating procedures (IdexxSA-AP-SOP-27). Subsequently, 40 µm transverse sections were cut at a thickness of 5 µm (IdexxSA-AP-SOP-30), mounted onto slides, and stained in an automated hematoxylin and eosin tissue stainer (IdexxSA-AP-SOP-205) before histological evaluation.

### 4.16. Statistical Analysis

The numerical values for each set of data are shown as mean ± standard deviation (STDEV). For the statistical significance test, the value of *p* ≤ 0.05 was considered statistically significant.

## 5. Conclusions

The main focus of this research article was to assess the potential of the silica gel (inorganic filler) reinforced 3D-printed biomaterial scaffold in vivo to activate host cells for potential complete bone critical-sized defect (5 mm diameter defect) healing and compared with an empty defect. Furthermore, BMP-7 (protein) was attached to the surface of the 3D-printed biomaterial scaffold as a trigger for osteogenic differentiation. Hence, the study assessed the potential of a silica-reinforced 3D-printed biomaterial scaffold (without BMP-7), a silica-reinforced 3D-printed biomaterial scaffold (with BMP-7), and an empty defect in assisting the host cells to heal critical-sized defects to completion.

Animal models have played indispensable roles over the years in the evaluation of biomaterial scaffolds for bone tissue engineering application and in understanding their regenerative potential and interaction with host bone tissue. Therefore, in our study, the 3D-printed biomaterial scaffold proved to be a potential boost for activation of host-cell self-healing more prominently compared to the empty similar size defect; however, the attachment of BMP-7 was found to be more prominent in activating the host-cells to self-heal to completion as compared to both the empty scaffold and the scaffold without BMP-7. Both 3D-printed biomaterial scaffolds induced no adverse tissue reaction and effectively supported and acted as a platform for host cells to adhere, proliferate, differentiate, and form new bone tissue in vivo. This indicates that the composite 3D-printed biomaterial scaffold containing BMP-7 can be a beneficial implant for bone tissue regeneration to be introduced for clinical tests. Therefore, our study confirmed the healing potential of the implant through the 5 mm diameter induced critical-sized nasal bone defects in rabbits.

## Figures and Tables

**Table 1:** The comparison of Young’s modulus of the three different printed scaffolds.

Bio-Inks	Solidification Solution	Young’s Modulus
Alg-PEI/Si 3D biomaterial scaffold	PEI dissolved in ethanol	60 MPa
Alg-PEI/HAP 3D biomaterial scaffold	PEI dissolved in ethanol	10 MPa
Alg-PEI/Nanoclay 3D biomaterial scaffold	PEI dissolved in ethanol	30 MPa

**Table 2:** Modified parameters were used for the 3D printing of the biomaterial scaffolds.

Parameters	Values
Temperature	37 °C
Pressure	0.8 bar
Speed	10.1 mm·s^−1^
Layers	18 layers
Platform temperature	37 °C
Preflow delay	0.15
Waiting time between layers	30 s

## Data Availability

Data presented are available upon request to the corresponding author.
